# Supplementary material for: Impact of moisturizing pretreatments on small reusable dental instruments cleanliness and mechanics
Source: PLoS One. 2026 Apr 29;21(4):e0348177. doi: 10.1371/journal.pone.0348177 (PMC13127945; doi:10.1371/journal.pone.0348177)
Supplement: Attachment — Submitted filename: Response to Reviewers.docx [file pone.0348177.s003.docx]

Dear Ph.D. Rawaa A. Faris，

We would like to express our sincere thanks to the reviewers for the constructive and positive comments. The detailed responses to the reviewers' comments are as follows.

According to the requirements of the journal, we have made corresponding checks and made the following revisions.

1. Our manuscript was completed in accordance with the formatting requirements of PLOS ONE's style.

2. The amended Funding Statement was added in cover letter.

3. The Wuhan University Clinical Nursing Special Research and Cultivation Fund Project (1607/600400021) was the only funder. The funder had no role in study design, data collection and analysis, decision to publish, or preparation of the manuscript.

4. All raw data required to replicate the results of our study was uploaded as supporting information in the file named “Data”.

5. I have an ORCID iD (0009-0002-789-9798), and also updated my information.

6. According to the requirements, we have removed the funding information in the Acknowledgments Section.

7. All figures were removed from revised manuscript file, and separately uploaded the individual TIFF/EPS image files.

8. Our reference was reviewed and it is complete and correct. No retracted paper was cited in our paper.

Replies to Reviewer #1

• *Authors should check for writing and typing errors.*

--According to the comment raised by the reviewer, we had checked the spelling and grammar in writing, and highlight with yellow.

• *The statements in discussion are acceptable but few paragraphs about the justification of your findings and comparison with other recent relevant studies.*
--We made corresponding modifications to the discussion and references section. We added two new paragraphs form line 276 (Page 10) to line 298 (Page 11). Minor enhancement was made from line 264 – 267 in Page 10. The integration was added from line 323 – 324 in Page 12.

• *Only include current up to date references in the reference list and remove outdated ones.*

--We deleted some outdated references (number 5, 8,17 and 25 in the original version), and added some current references (number 22 - 29 in revised manuscript and marked with red).

Replies to Reviewer #2

• *The title should be revised and reduced its characters.*

--We revised and reduced the title characters and marked with red.

• *Four to six keywords representing the main content of the article BUT not mentioned in the title.*

-- We have revised some keywords and marked with red.

• *More paragraphs should be incorporated to introduction and discussion although the statements in discussion are acceptable but few paragraphs about the justification of your findings and comparison with other recent relevant studies.*

-- This opinion is similar to the second question raised by Reviewer #1. It has already been addressed previously.

• *All* *numerical values and percentage should be omitted from conclusion section.*

--There was no numerical values and percentage in conclusion section.

• *The author should pay attention to writing and typing errors throughout the manuscript.*

-- we had checked the spelling and grammar in writing, and highlight with yellow.

• *Up to date references should be kept in your reference list and the old should be omitted.*

--According to the comment raised by the reviewer, we had deleted the outdated references.

Sincerely

Tianjuan Xia
