# Supplementary material for: Impact of moisturizing pretreatments on small reusable dental instruments cleanliness and mechanics
Source: PLoS One. 2026 Apr 29;21(4):e0348177. doi: 10.1371/journal.pone.0348177 (PMC13127945; doi:10.1371/journal.pone.0348177)
Supplement: Attachment — Submitted filename: Response_to_Reviewers_auresp_2.docx [file pone.0348177.s004.docx]

Dear Ph.D. Rawaa A. Faris，

We would like to express our sincere thanks to your opinion about our manuscript. The detailed responses to your comments are as follows.

*If the reviewer comments include a recommendation to cite specific previously published works, please review and evaluate these publications to determine whether they are relevant and should be cited. There is no requirement to cite these works unless the editor has indicated otherwise.* 
--- The reviewers did not recommend any papers for citation.

*Please review your reference list to ensure that it is complete and correct. If you have cited papers that have been retracted, please include the rationale for doing so in the manuscript text, or remove these references and replace them with relevant current references. Any changes to the reference list should be mentioned in the rebuttal letter that accompanies your revised manuscript. If you need to cite a retracted article, indicate the article’s retracted status in the References list and also include a citation and full reference for the retraction notice.*
---The references were checked and revised. The revised references were marked with red.

---The reference [5] was updated. The old one “Sterilization of health products in public services (2016)” was replaced by the new one “Australian Dental Association (2024) Guidelines for Infection Control (2024). Fifth Edition”.

--- The old title was wrong in the reference [18]. The corrected title “The Role of ATP Luminometers in Infection Control” was marked with red.

---The time was wrong in the reference [19]. The correct time is 2013.

---No retracted paper was cited in our manuscript.

*While revising your submission, please upload your figure files to the Preflight Analysis and Conversion Engine (PACE) digital diagnostic tool,* [*https://pacev2.apexcovantage.com/*](https://pacev2.apexcovantage.com/)*. PACE helps ensure that figures meet PLOS requirements. To use PACE, you must first register as a user. Registration is free. Then, login and navigate to the UPLOAD tab, where you will find detailed instructions on how to use the tool. If you encounter any issues or have any questions when using PACE, please email PLOS at* [*figures@plos.org*](mailto:figures@plos.org)*. Please note that Supporting Information files do not need this step.*

---The PACE corrected figures were obtained through the Preflight Analysis and Conversion Engine (PACE) digital diagnostic tool, and re-uploaded.

Sincerely

Tianjuan Xia
